# Comparing the Efficacy of Anodal, Cathodal, and Sham Transcranial Direct Current Stimulation on Brain-Derived Neurotrophic Factor and Psychological Symptoms in Opioid-Addicted Patients

**DOI:** 10.32598/BCN.10.6.1710.1

**Published:** 2019-11-01

**Authors:** Zakaria Eskandari, Mohsen Dadashi, Hossin Mostafavi, Alireza Armani Kia, Reza Pirzeh

**Affiliations:** 1. Department of Clinical Psychology, Faculty of Medicine, Zanjan University of Medical Sciences, Zanjan, Iran.; 2. Department of Clinical Psychology, Faculty of Medicine, Social Determinants of Health Research Center, Zanjan University of Medical Sciences, Zanjan, Iran.; 3. Department of Clinical Psychology, Social Determinants of Health Research Center, Zanjan University of Medical Sciences, Zanjan, Iran.; 4. Department of Physiology, Faculty of Medicine, Zanjan University of Medical Sciences, Zanjan, Iran.

**Keywords:** Transcranial direct current stimulation, Craving, Opioid, Brain-derived neurotrophic factor, Depression dorsolateral prefrontal cortex, Anxiety

## Abstract

**Introduction:**

Today, addiction to opioids is a serious problem all over the world. Unfortunately, the consumption of these drugs and the number of addicted people have drastically increased. This research aimed at comparing the efficacy of anodal, cathodal, and sham transcranial Direct Current Stimulation (tDCS) on the Brain-Derived Neurotrophic Factor (BDNF) and psychological symptoms in opioid-addicted patients.

**Methods:**

Thirty opioid-addicted patients were selected based on the Diagnostic and Statistical Manual of Mental Disorders, the Fifth Edition, through the convenience sampling method. They were then randomly assigned to 3 groups (10 in each group). The subjects were evaluated before and after tDCS by their serum level of BDNF, desires for drug questionnaire, and depression anxiety stress scale. The data were analyzed by the Kolmogorov-Smirnov test, one-way analysis of variance, as well as the Bonferroni test.

**Results:**

Stimulating the Dorsolateral Prefrontal Cortex (DLPFC) led to a significant change in increasing the level of BDNF (P=0.031) and reducing the degree of depression (P=0.018), anxiety (P=0.001), stress (P=0.012), and decreased the level of craving (P=0.001) in opioid-addicted patients. There was no significant difference between active stimulation groups (anodal left/cathodal right and anodal right/cathodal left). The stimulation of the right DLPFC (group B) significantly increased BDNF in comparison with the sham group (sham tDCS) and decreased anxiety and craving. Nonetheless, no change was observed in depression and stress. The stimulation of the left DLPFC (group A) significantly reduced depression, anxiety, stress, and craving compared with the sham group, while there was no change in BDNF.

**Conclusion:**

In addition to the conventional treatments of opioid-addicted patients, tDCS is an effective complementary treatment.

## Highlights

- Stimulating the dorsolateral prefrontal cortex can significantly increase the Brain-Derived Neurotrophic Factor (BDNF) and decrease the symptoms of depression, anxiety, stress, and craving.
- The stimulation of the right frontal area can increase BDNF serum levels.
- The stimulation of bilateral brain regions can reduce the symptoms of anxiety, depression, and craving.

## Plain Language Summary

Opioid addiction is one of the main concerns of societies and is considered a cerebral chronic relapsing disease. In spite of its negative consequences, opioid addiction is wildly common. The Brain-Derived Neurotrophic Factor (BDNF) increases the growth, survival, and the health of different neurons. It is considered an essential adjusting factor of brain flexibility. Drug taking changes the expression of endogenous BDNF neuron circuits responsible for the addictive behaviors. According to the studies, the higher expression of BDNF can neutralize the effects of taking opioids. This research aimed to study the effectiveness of stimulating the DLPFC with two protocols of stimulating anodal right/cathodal left and sham Transcranial Direct Current Stimulation (tDCS) in the Dorsolateral Prefrontal Cortex (DLPFC) area to increase the BDNF and reduce the level of depression, anxiety, stress, and craving for drug taking. Thirty opioid-addicted patients were selected by sampling through the web and were divided into 3 groups (10 individuals in each group). Group A received the anodal right/cathodal left stimulation, group B anodal left/cathodal right stimulation, and group C received the sham stimulating. Stimulating the DLPFC utilizing two real and active protocols had the same effects and there was no significant difference between them, but group B (anodal right/cathodal left) versus group C (sham-tDCS) could significantly increase the level of the BDNF and decrease the craving. Therefore, brain-stimulating can be considered an alternative for the treatment of opioid-addicted patients. The BDNF can be used as a biomarker responding to the treatment.

## 1. Introduction

Today, opioid addiction is considered the main concern for all nations because of the high rate of drug abuse and addiction to them. Addiction to narcotic drugs is connected to social problems such as unemployment, legal issues, and interpersonal problems, as well as health problems such as HIV and death. Because of these reasons, addiction to opioids is among chronic relapsing diseases (Veilleux et al., 2010).

An important matter in addiction treatment is the mental illnesses (that are the groundwork of mental disorders) as a consequence of abstinence as they can result in treatment failure. Many studies have emphasized the outbreak of mental disorders such as anxiety and depression in patients addicted to narcotics (Tabatabaei Chehr, Ebrahimi Sani, & Mortazavi, 2012).

The chronic drug taking can cause neurochemical changes in higher areas of the cortex. These changes make the drug fascinating to the users and weaken the strength of avoiding drugs. These changes can explain the appearance of an intense craving for taking drugs and keeping on taking it in spite of its adverse effects. The neuroadaptation in other areas of the frontal cortex plays a role in an inability to understand the consequences of drug taking. The studies in neuroscience suggest that addiction is a kind of sickness type behavior that affects the natural process of learning and motivation in the brain so that taking drugs overcomes any other rewarding behavior. Knowing this view makes it easier to find new and more effective ways of addiction treatment. It makes these changes a target and also causes changes in attitudes and treatment methods of addiction (Carter, Hall, & Nutt, 2009).

Since the advent of psychopharmacologic treatments in the early 1950s and after the development of the anti-depression medicines of the second generation and anti-psychotic medication, biological psychiatry has had little achievements. In the light of recent technological advancements in non-invasive brain-stimulating, new horizons have appeared in the treatment of psychological disorders. Among these, transcranial direct current stimulation (tDCS) can adjust the cortical excitability and has long-term effects. Applying the tDCS can be suitable for the patients, who report side effects of taking medicines or the patients who show resistance to the medical symptoms. The number of studies on the impact of this treatment on psychological disorders has increased in recent years (Mondino et al., 2014).

In spite of considerable advances in the field of medicinal and non-medicinal treatments for drug-taking disorders, these treatments for addiction show some limitations that emphasize the need for new alternatives in treatment (Lupi et al., 2017). New ways in treatment, such as non-invasive brain stimulation, have been developed in the domain of drug-taking disorders. One of these treatments is tDCS (Sauvaget et al., 2015).

Generally, anodal tDCS depolarizes neurons, thereby increasing cortical excitability, whereas cathodal tDCS hyperpolarizes neurons, diminishing cortical excitability (Nitsche & Paulus, 2001; Stagg & Nitsche, 2011). Glutamatergic mechanism mediates the long-term effects of tDCS on cortical excitability (Liebetanz, Nitsche, Tergau, & Paulus, 2002; Nitsche et al., 2006). The brain-derived neurotrophic factor (BDNF) is the most important neurotrophin. It increases the growth, survival, and the health of different neurons, and it is also an important modulating factor of brain flexibility. In other words, the release of BDNF in synapses increases the synaptic transfer and the neuron stimulating, and in turn, the behavioral and learning stimulation increases the gene expression of BDNF (Mooren & Volker, 2005).

Drug addiction causes some changes in the expression of endogenous BDNF neuron circuits responsible for addictive behaviors. BDNF has been recognized as a mediator of memory consolidation in different behavioral and neuro-physiological levels. Special neuron circuits are responsible for the storing and running of the food receiving movement programs. On the other hand, the other neuron circuits are responsible for the active repression of these food receiving systems (Barker, Taylor, De Vries, & Peters, 2015).

The increase of BDNF expression can neutralize the effect of taking opioids on neurons for a long time. Studies on animals have indicated that long-term taking of opioids brings biochemical and morphological changes in the ventral tegmental area, and some of these changes can be prevented happening by injecting BDNF into this area of the brain (Berhow et al., 1995; Sklair-Tavron et al., 1996).

The results of the studies showed that the increase of serum level BDNF could be connected to the pathophysiology of addiction to opioids and withdrawal signs. Yet, there is a need for longer follow-up studies to determine the role of the BDNF as a potential biologic biomarker in addition to the opioids and the signs of the withdrawal (Zhang et al., 2014).

It is assumed that the BDNF shows the mental status, and meta-analysis indicates that it can show the mood of an individual (Fernandes et al., 2015). The higher the level of BDNF, the better the cognitive functioning (Bekinschtein, Oomen, Saksida, & Bussey, 2011; Novkovic, Mittmann, Manahan & Vaughan, 2015). This research studied the degree of BDNF serum level changes resulting from the tDCS.

Therefore, the present study aimed at investigating whether there is a difference between the two tDCS protocols in changing BDNF serum level and reducing the level of depression signs, anxiety, stress, and craving for drugs among opioid-taking patients.

## 2. Methods

### 2.1. Study procedure

The present quasi-experimental research used a pre-test-post-test design administered on 3 groups. The statistical population included all opioid-addicted patients in Zanjan City, Iran. A sample of 30 patients was selected through convenience sampling method. They were then randomly assigned to 3 groups (10 individuals in each group). The inclusion criteria included 1. Giving a conscious consent for participation; 2. Having a history of taking opioids and its derivatives; 3. Being under the methadone treatment for at least 2 weeks; 4. Being male, 5. Being 18–50 years old, and 6. Passing at least secondary high school. The exclusion criteria included 1. Being absent for 2 sessions from the intervention; 2. Having a risk of committing suicide ideation (making it impossible for researchers to have the medication dosage fixed); 3. Having severe mental disorders such as schizophrenia, 4. Taking several narcotic drugs together; 5. Having a history of damage on head, and 6. Having a history of epileptic seizure. After filling out a consent form, they were evaluated by the ELISA technique to measure their serum level of BDNF. Two questionnaires were administered namely Desires for Drug Questionnaire (DDQ) and Depression Anxiety Stress Scale (DASS). Then, tDCS was applied for the following groups in 10 20-minute-sessions: group A. L-dorso-lateral prefrontal cortex (DLPFC) anodal left/cathodal right; group B. R-DLPFC anodal right/cathodal left; and group C. Sham-tDCS. In direct current stimulating over the cortex treatment, 2 electrodes were placed on the head, one of which with a positive pole and the other with a negative pole. The electrodes were moistened by a sponge pad in a conductive solution. After passing through different areas (head skin, skull, etc.), the electric current met the cortex. The current reaching this area charged the neurons and created positive/negative poles, which changed the activities in that area. Based on the special disorder, the amperage, duration, direction, the place of pads, the size of pads, and the number of sessions were considered. After the intervention, the serum level of BDNF was measured and the questionnaires were filled out again. The data were presented with descriptive statistics (Mean±SD, frequency, and tables). The Kolmogorov-Smirnov test was used for determining the normality and Levene’s test was used for examining the variance uniformity. Also, one-way variance analysis and the Bonferroni tests were used. The data were analyzed in SPSSS V. 22.

### 2.2. Study instruments

#### 2.2.1. Transcranial Direct Current Stimulation

In tDCS, a weak direct current in the ranged from 0.5–2 mA is generated. The electrodes are directly connected to the head with a pair of electrode pads moistened with saline. In tDCS treatment, 2 electrodes are placed on the head, one with a positive pole and the other with a negative pole moistened by a sponge pad in a conductive solution (Spagnolo & Goldman, 2016).

#### 2.2.2. Desires for Drug Questionnaire

Franken et al., measured the urgent or instantaneous desire of the patient to take drugs. The Desires for Drug Questionnaire (DDQ) was first drawn up for measuring the craving for alcoholic drinks on alcoholic patients. Thanks to its general structure, it can be used for measuring craving for other cases of addiction. Frankn et al., used this questionnaire to measure craving for heroin and, then, recommended it in any case of addiction by making a little modification. The validity and internal reliability of this questionnaire were tested on 102 Dutch patients who were addicted to drug treatment. Their study on heroin takers showed desirable reliability and validity. It can be used in other fields of clinical research, too.

There are 14 items in this questionnaire. It is scored from 0–10, and the higher scores indicate higher craving. This questionnaire includes 3 elements of “desire and tendency”, “negative reinforcement”, and “control” and their Cronbach alpha values (for measuring the internal consistency) were 0.81, 0.82, and 0.79, while their test-retest results for each element were 0.83, 0.82, and 0.74, respectively (Franken, Hendriks, & Van den Brink, 2002). Also, the common variance of 3 elements was 0.62.

#### 2.2.3. Depression Anxiety Stress Scale

Lavvibavnd and Lavibvand designed this 21-item scale. DASS-21 has three subscales of depression, anxiety, and tension, 7 items each. The total score is acquired by adding the scores of each subscale. Every item is scored from 0 (it is not true for me) to 3 (It is completely true for me). Higher scores indicate low mental health. In Iran, Samani and Jokar retested the scale. The validity coefficient was 80%, 76%, and 77% for depression, anxiety, and stress, respectively. The Cronbach alpha values were found 0.81, 0.74, and 0.75 for depression, anxiety, and stress, respectively (Samani & Jokar, 2007).

## 3. Results

The study groups were homogenous, with no significant difference between them considering their age and duration of illness (Table 1). Table 2 presents the mean and SD of scores of patients in research variables in pre-test and post-test stages. Table 3 presents the results of the scores of variables in the pre-test and post-test. According to Table 4 and regarding the mean differences of the research variables, we can observe a statistically significant difference. Regarding no statistically significant difference between the means of the groups considering these variables before the intervention, we could draw this conclusion that is homogenous.

**Table 1.** One-way analysis of variance for variables of age and duration of illness

**Variables**		**df**	**Sum of Squares**	**Mean of Squares**	**F**	**P**
Age	Between groups	2	0.996	0.498		
	Within groups	28	2312.682	82.596	0.006	0.994
	Total	30	2313.677			
Illness duration	Between groups	2	1.990	0.995		
	Within groups	28	941.945	33.641	0.030	0.971
	Total	30	943.935			

**Table 2.** Research variables in the pre-test and post-test presented

**Groups**	**Stages**	**Mean±SD**				

		**BDNF**	**Depression**	**Anxiety**	**Stress**	**Craving**
Group A ^*^	Pre-test	6.08±4.11	28.20±8.66	24.60±7.77	30.80±6.94	68.80±31.19
	Post-test	6.63±5.39	14.60±8.94	9.60±6.97	17.20±11.40	14.20±10.89
Group B ^**^	Pre-test	5.88±4.22	29.45±6.99	24.73±8.86	31.82±8.07	75.73±26.06
	Post-test	7.62±5.48	17.09±9.48	11.82±7.40	20±7.74	28.27±18.69
Group C ^***^	Pre-test	2.89±0.89	26.20±7.51	24±7.95	31.60±5.06	75.70±25.59
	Post-test	2.37±0.87	26.80±9.94	23.20±8.65	29.60±7.58	63.30±29.19

* Anodal left/cathodal right;

** Anodal right/cathodal left;

*** Sham tDCS

BDNF: Brain-Derived Neurotrophic Factor

**Table 3.** One-way analysis of variance results of the scores of variables in pre-tests

**Variables**		**df**	**Sum of Squares**	**Mean of Squares**	**F**	**P**
BDNF	Between groups	2	64.44	32.22		
	Within groups	28	337.48	12.05	2.67	0.87
	Total	30	401.92			
Depression	Between groups	2	56.07	28.03		
	Within groups	28	1671.92	59.71	0.47	0.63
	Total	30	1782			
Anxiety	Between groups	2	29.01	14.50		
	Within groups	28	1866.98	66.67	0.21	0.80
	Total	30	1896			
Stress	Between groups	2	5.91	2.95		
	Within groups	28	1315.63	46.98	0.06	0.93
	Total	30	1321.54			
Craving	Between groups	2	323.86	161.93		
	Within groups	28	21445.88	65.92	0.21	0.811
	Total	30	21769.74			

BDNF. Brain-Derived Neurotrophic Factor

**Table 4.** One-way analysis of variance results of the scores of variables in post-tests

**Variables**		**df**	**Sum of Squares**	**Mean of Squares**	**F**	**P**
BDNF	Between groups	2	159.62	79.81		
	Within groups	28	569.45	20.33	3.92	0.031
	Total	30	729.081			
Depression	Between groups	2	836.63	418.32		
	Within groups	28	2508.90	89.60	4.66	0.018
	Total	30	3345.54			
Anxiety	Between groups	2	1073.78	536.89		
	Within groups	28	1659.63	59.27	9.05	0.001
	Total	30	2733.41			
Stress	Between groups	2	850.83	425.41		
	Within groups	28	2288.000	81.71	5.20	0.012
	Total	30	3138.83			
Craving	Between groups	2	12833.08	6416.54		
	Within groups	28	12235.88	436.99	14.68	0.000
	Total	30	25068.96			

BDNF. Brain-Derived Neurotrophic Factor

After the intervention, there was a significant difference between the treatment groups in increasing the expression level of BDNF (P=0.031), decreasing craving (P=0.000), and decreasing the symptoms of depression (P=0.018), anxiety (P=0.001), and stress (P=0.012).

There is a significant difference between the groups (Table 4). Thus, the researchers administered a post hoc test (Table 5). The results of paired comparisons suggest that concerning BDNF in group B vs. group C, the difference was significant (P=0.042). Regarding depression in group A vs. group C, we can see a significant difference (P=0.023). Also, we can observe a significant difference in anxiety in group A vs. group C and group B vs. group C, (P=0.001 and P=0.006). Concerning stress in group A vs. group C, there was a significant difference (P=0.014). Likewise, regarding craving, there were significant differences between group A vs. group C and B vs. group C (P=0.000 and P=0.002). Table 6 presents the mean of baseline (pre-test), post-test, and percentage of change in the intervention groups and the variables.

**Table 5.** Bonferroni test results comparing the paired variables of BDNF, depression, anxiety, stress, and craving in the post-test

**Dependent Variables**	**Group A vs. B**		**Group A vs. C**		**Group B vs. C**	

	**Mean Differences**	**P**	**Mean Differences**	**P**	**Mean Differences**	**P**
BDNF	−0.99	0.882	4.252	0.12	0.247	0.042
Depression	−2.49	1	−12.20	0.023	−9.70	0.079
Anxiety	−2.21	1	13.60	0.001	−11.38	0.006
Stress	−2.80	1	−12.40	0.014	−9.60	0.065
Craving	−14.07	0.404	−49.10	0.000	−35.02	0.002

BDNF. Brain-Derived Neurotrophic Factor

**Table 6.** The change of variables in the groups compared with the baseline and percentage of changes

**Variables**	**Group A**			**Group B**			**Group C**		

	**Pre-test**	**Post-test**	**%**	**Pre-test**	**Post-test**	**%**	**Pre-test**	**Post-test**	**%**
BDNF	6.08	6.63	8.29	5.88	7.62	22.83	2.89	2.37	−21.94
Depression	28.20	14.40	48.93	29.45	17.09	41.96	26.20	26.80	−2.29
Anxiety	24.60	9.60	60.97	24.73	11.82	52.20	24	23.20	3.33
Stress	30.80	17.20	44.15	31.82	20	37.14	31.60	29.60	6.32
Craving	68.80	14.20	79.36	75.73	28.27	62.67	75.70	63.30	16.38

BDNF: brain-derived neurotrophic factor

The percentage of change in BDNF was 8.29%, 22.83%, and −21.94% in groups A, B, and C, respectively. Although BDNF changed in both A and B groups, it significantly changed in group B. In group C (sham), it decreased. The percentage of change in depression was 48.93%, 41.96%, and −2.29% in groups A, B, and C, respectively. Although depression changed in both A and B groups, the change was slightly higher in group B. In group C, it slightly decreased. The percentage of change in anxiety was 60.97%, 52.20%, and 3.33% in groups A, B, and C, respectively. Although anxiety changed in both A and B groups, it was relatively more in group A. In group C, the change was negligible. The percentage of changes in stress was 44.15%, 37.14%, and 6.32% in groups A, B, and C, respectively. Although stress changed in both A and B groups, it was relatively more in group A. In group C, and the change was trivial. The percentage of s in craving was 79.36%, 62.67%, and 16.38% in groups A, B, and C, respectively. Although craving changed in groups A and B, it changed relatively more in group A. In group C, the change was negligible.

## 4. Discussion

This research aimed to find out the effect of tDCS over DLPFC by measuring the serum level of BDNF and the decrease of the craving and psychological symptoms in opioid-addicted patients. Administrating tDCS over the cortex increases the level of BDNF and decreases the psychological symptoms such as depression, anxiety, stress, and craving for drugs. The intervention had a significant impact on the variables of the research. Furthermore, the paired comparisons of the research variables suggested that in groups A and B, a real and active stimulation with an equal size of impact was exercised, and there was no significant difference between the two groups. Regarding BDNF, the difference was not significant between groups A and C. Nonetheless, there was a significant difference in depression, anxiety, stress, and craving between two groups.

There was also a significant difference in the BDNF blood level of patients taking opioids, who received tDCS. But, we noticed that only group B vs. group C, which did not receive a real stimulation, could significantly increase the level of BDNF in blood. We could not find any similar study on this issue in the literature. Therefore, this research is not comparable with the previous ones. Since this research was an introductory one, it can open new doors to future research. However, we can review some studies carried out merely on the relationship between BDNF and opioids.

It seems that neuroplasticity changes and cortex excitability are important pathophysiological factors in many neuropsychological illnesses, including addiction to narcotic drugs. Therefore, non-invasive brain stimulation can be a valuable approach for changing and modifying cortex activities (Lefaucheur et al., 2017). So far, the two study results dealt with the topic of BDNF serum level among the heroin takers are contradictory. Angelucci et al., (2007) found a reduction in BDNF serum levels in patients addicted to heroin. However, Heberlein et al., showed an increase in BDNF serum level in patients addicted to heroin, substituted by opioids (Heberlein et al., 2011). The reasonably small sample size (N=15) in the case of Angelucci et al., can be blamed for their results. In addition, nicotine and alcohol (Joe et al., 2007), depression (Molendijik et al., 2011), and stress (Miltoma et al., 2008) can play a role in obtaining the different results.

The results of Heberlein et al., study showed that the BDNF and the glial cell line-derived neurotrophic factor (GDNF) are involved in adjusting addictive behaviors. While studying the serum level of BDNF and GDNF in their patients addicted to opioids, they showed that BDNF serum level had a significant difference in patients addicted to opioids under the treatment of diacetylmorphine in an opiate maintenance program compared to a healthy control group. However, the GDNF serum level did not show a significant difference (Heberlien et al., 2011).

Opioids poison the central nervous system that is related to the changes in BDNF expressions. Hence, the environmental basic BDNF level in opioid abuse disorder patients can be changed or be modified by avoiding taking drugs (Palma-Alvarez et al., 2017). Heberlien et al., (2011) reported that patients cured by diacetylmorphine had a higher level of BDNF in their sample serums, while Lee et al., showed no change in the plasma BDNF concentration of the opioid abuse disorder patients (Lee et al., 2015).

The study by Zhang et al., (2016) indicated that the BDNF serum level in the baseline significantly was lower than that of the control group of heroin-addicted patients. Besides, a significant difference was observed in the BDNF serum level in patients addicted to heroin in baseline and 26 follow-up sessions. BDNF serum level was not related to the age, body mass index, education, and the age of starting drug-taking or duration of the taking drugs (Zhang et al., 2016). Such findings are congruent with the result of this research, in particular regarding the effectiveness of BDNF using the general treatment and cortex stimulation. There was a statistically significant difference in depression in the opioid patients, who received tDCS. However, we found merely a significant difference between groups A and C.

Until now, all studies have investigated the anodal stimulation DLPFC left and cathodal controlling the right DLPFC in basic depression disorder (Dunlop, Hanlon, & Downar, 2017). A few studies have studied the effects of tDCS on the moods of participants with addiction disorders (Kekic, Boysen, Campbell, & Schmidt, 2016). In the present study, group A that received anodal left, and cathodal right stimulation had a significant difference with group C in reducing depression; but, group B that received right anodal and left cathodal stimulation did not show a significant difference in lowering depression compared to group C.

Generally, the effects of tDCS on the mood seem to be independent of the impact of searching and taking drug behaviors. Further studies are warranted to explore the effects of potential mood-changing tDCS on participants affected by addictive disorders, as well as the comorbidity of depression disorders or anxiety (Spagnolo & Goldman, 2016).

There was a significant difference in the anxiety level of opioid-addicted patients who received tDCS. Nonetheless, there was no significant difference between groups A and B, and both groups had a significant difference in relation to group C. In other words, the two protocols of brain stimulation could reduce the patient’s anxiety. These findings are consistent with those reported in the studies of de Almeida Ramose, Taiar, Trevizol, Schiozawa and Cordeiro (2016), Batista, Klauss, Fregni, Nitche and Nakamura-Palacios (2015) and Hashemi, Nazari, Yassini, & Mirhosseini (2015) that reported a reduction in the level of anxiety in drug-taking people.

There was a significant difference in the stress of opioid-addicted patients, who received tDCS, but there was no significant difference between groups A and B, and both groups had a significant difference compared to group C. This implies that the two protocols of brain stimulation can reduce patient’s stress. The results are in line with those of Moradi, Kelardeh, Yaryari, and Abdollahi (2016) that reported a downfall in the stress levels of drug-taking patients utilizing tDCS.

There was a significant difference in the drug craving of opioid-addicted patients who received tDCS. However, the results of the Bonferroni test did not show a significant difference between groups A and B, and both groups had a significant difference compared with group C. Similarly, the two protocols of brain stimulation could reduce the opioid-addicted patients craving for drugs.

As far as the probable mechanisms active in the aftereffects reported following tDCS sessions, the following postulations could be raised and discussed. From a pharmacological perspective, the after-effects of anodal tDCS hinge on the polarization of the membrane. The application of a calcium or sodium channel blocker terminated the after-effects of tDCS. Furthermore, dextrorphan (antagonist of the N-methyl-D-aspartate [NMDA] receptor) was reported to impede the induction of long-term after-effects generated by tDCS, regardless of polarity (Liebetanz et al., 2002). Most likely, such findings indicate that tDCS triggered after-effects depending on the modification of NMDA-receptor sensitivity. Dopaminergic receptors take part in NMDA-receptor-dependent neuroplasticity. Nitsche et al., (2006) reported that the obstruction of D2 by sulpiride suppresses the enactment of the after-effects through tDCS. Such a finding confirms the vital role of the NMDA receptor in the observable after-effects following a tDCS session. Likewise, some other studies have revealed that tDCS triggers plastic changes comprised of regulation of a wide range of other neurotransmitters, such as dopamine, acetylcholine, and serotonin. An extensive number of alterations could be triggered at diverse levels on the part of a weak DC stimulation. Additional confirmatory studies are warranted to fathom better a host of mechanisms verifying tDCS after-effects. Their findings could be employed to improve such after-effects clinically (Roche, Geiger, & Bussel, 2015).

The reason for the use of tDCS in treating drug disorders and craving is that DLPFC plays an essential role in controlling top-down inhibition mechanism and rewarding mechanism, which is presumably disturbed in these kinds of disorders (Lefaucheur et al., 2017). This finding is in line with those of Wang et al., (2016), Basista et al., (2015), that reported a reduction in craving through tDCS.

## 5. Conclusion

As the limitations of this research, we could not follow the investigation at least for 6 months, the participants were male, and the size of the sample was small. Therefore, it is suggested that future studies be conducted with a 6-month follow-up regarding the consistency and sustainability of serum level BDNF and changes of psychological symptoms. Also, reducing the craving level and including female participants with more population in the study are suggested in future studies.

## Ethical Considerations

### Compliance with ethical guidelines

This study was conducted in compliance with the ethical guidelines of Zanjan University of Medical Sciences. The ethics code No IR.ZUMS.REC.1397.127 was issued for this study, and it was registered in the Iranian Registry of Clinical Trials with IRCT IRCT20170513033946N5. The Local Ethics Committee of Zanjan University of Medical Sciences approved the research. Before participation in the study, all participants provided informed written consent.
